# Noninvasive ventilation in patients with acute respiratory distress syndrome

**DOI:** 10.1186/s13054-019-2666-4

**Published:** 2019-11-15

**Authors:** Kailei Du

**Affiliations:** Intensive Care Unit, Affiliated Dongyang Hospital of Wenzhou Medical University, NO. 60 Wuning West Road, Dongyang, 322100 China

To the Editor:

The benefits of noninvasive ventilation (NIV) in acute respiratory distress syndrome (ARDS) have long been debated. In a recent well-designed trial, He et al. [1] found that compared to standard oxygen therapy, NIV in mild ARDS patients did not reduce the intubation rate. We would like to add some comments. First, even though a targeted tidal volume (*V*_T_) was set, adequate control of the *V*_T_ was difficult during NIV. In an observational study [2], Carteaux et al. reported that despite setting the targeted *V*_T_ at 6–8 mL/kg, the actual *V*_T_ was 9.8 mL/kg during application (75% of patients had *V*_T_ > 8 mL/kg). Similarly, the LUNG-SAFE study [3] also reported that the mean *V*_T_ was 8.73 mL/kg in ARDS patients managed with NIV. Physiologically, *V*_T_ during NIV is the consequence of both the ventilation-inspiratory pressure support and the spontaneous respiratory muscle activity. Thus, even with the inspiratory pressure set at a low level, patients may still spontaneously breathe with a high *V*_T_ because of a strong respiratory effort to alleviate tachypnea/dyspnea. Second, differences in ARDS severity remain an important cause of inconsistent findings. In a propensity-matched analysis, Bellani et al. [3] found that in a group of ARDS patients with PaO_2_/FIO_2_ < 150 mmHg, the mortality of the NIV group was significantly higher than that of the invasive ventilation group. This may be due to the fact that tidal hyperinflation during NIV may be more significant as the lung in patients with severe ARDS is stiffer, which is a significant risk factor for ventilation-induced lung injury. In the study by He et al. [1], only patients with mild ARDS were investigated. However, Shen et al. [4] found that the benefit of low *V*_T_ ventilation is more significant in mild than in severe ARDS. Thus, whether NIV should be considered when treating ARDS still requires investigation. Third, there are different NIV approaches. In 2016, Patel et al. [5] showed that the use of helmet NIV resulted in a significant reduction in the rate of intubation when compared to mask (8/44 vs. 24/39, *p* < 0.001). However, in the helmet group, the positive end-expiratory pressure (PEEP) was significantly higher (8 [5.0–10.0] vs. 5.1 [5.0–8.0], *p* = 0.006) and the supported pressure was lower (8 [5.6–10.0] vs. 11.2 [10.0–14.5], *p* < 0.001) than in the mask group. Higher PEEP and lower pressure support are supposed to be associated with lower driving pressure. Thus, whether the benefit of helmet NIV was a result of a more protective ventilation strategy remains unclear. Finally, we suggest that further studies should incorporate mechanical indexes (low *V*_T_, driving pressure, etc.) into NIV application to obtain more positive results.

## Data Availability

Not applicable.
